# Influence of ZrO_2_ Addition on Structural and Biological Activity of Phosphate Glasses for Bone Regeneration

**DOI:** 10.3390/ma13184058

**Published:** 2020-09-12

**Authors:** M. Mohan Babu, P. Syam Prasad, P. Venkateswara Rao, S. Hima Bindu, A. Prasad, N. Veeraiah, Mutlu Özcan

**Affiliations:** 1 Department of Physics, National Institute of Technology Warangal, Warangal 506004, India; mmbabu771@gmail.com (M.M.B.); h.bindu05@gmail.com (S.H.B.); prasadbabunitw@gmail.com (A.P.); 2 Department of Physics, The University of the West Indies, Mona Campus, Kignston 7, Jamaica; pvrao54@gmail.com; 3 Department of Physics, Acharya Nagarjuna University, Nagarjuna Nagar, Guntur, AP 522510, India; profnvr@gmail.com; 4 Center for Dental and Oral Medicine, Division of Dental Biomaterials, Clinic for Reconstructive Dentistry, University of Zurich, 8032 Zurich, Switzerland; mutluozcan@hotmail.com

**Keywords:** P_2_O_5_-bioglass, zirconia, melt-quenching, SBF, hydroxyapatite, in vitro bioactivity

## Abstract

Zirconium doped calcium phosphate-based bioglasses are the most prominent bioactive materials for bone and dental repair and regeneration implants. In the present study, a 8ZnO–22Na_2_O–(24 − x)CaO–46P_2_O_5_–xZrO_2_ (0.1 ≤ x ≤ 0.7, all are in mol%) bioglass system was synthesized by the conventional melt-quenching process at 1100 °C. The glass-forming ability and thermal stability of the glasses were determined by measuring the glass transition temperature (*T*_g_), crystallization temperature (*T*_c_), and melting temperature (*T*_m_), using differential thermal analysis (DTA). The biological activity of the prepared samples was identified by analyzing X-ray diffraction (XRD), Fourier transform infrared spectroscopy (FTIR) and scanning electron microscopy-energy dispersive spectra (SEM-EDS), before and after immersion in simulated body fluid (SBF) for various intervals of 0, 1 and 5 days, along with the magnitude of pH and the degradation of glasses also evaluated. The obtained results revealed that the glass-forming ability and thermal stability of glasses increased with the increase in zirconia mol%. The XRD, FTIR, and SEM-EDS data confirmed a thin hydroxyapatite (HAp) layer over the sample surface after incubation in SBF for 1 and 5 days. Furthermore, the development of layer found to be increased with the increase of incubation time. The degradation of the glasses in SBF increased with incubation time and decreased gradually with the increase content of ZrO_2_ mol% in the host glass matrix. A sudden rise in initial pH values of residual SBF for 1 day owing to ion leaching and increase of Ca^2+^ and PO_4_^3−^ ions and then decreased. These findings confirmed the suitability of choosing material for bone-related applications.

## 1. Introduction

Bioactive glasses are the widely used surface reactive inorganic biomaterials in engineering, essentially for the repair and regeneration of damaged soft and hard bone tissues [1,2,3,4]. 45S5 bioglass is the well-known and widely used bioactive glass developed by Professor Larry Hench and his co-workers in 1969 [5]; comprised of inorganic oxides (viz., SiO_2_, Na_2_O, CaO, and P_2_O_5_) in a specific molar ratios and has exhibited thriving biological properties such as in vitro bioactivity, osteostimulative and osteoconductive properties. These special qualities made the bioglass a biocompatible and bioresorbable material in comparison to the natural bone. Various types of melt-derived bioactive glasses (SiO_2_, B_2_O_3_, P_2_O_5_ etc.) have been developed and tested because of their capability to interact with living bone tissues [3,6]. Out of all these glasses, phosphate-based glasses have many advantages, due to solubility, high biocompatibility and low melting temperature etc., which made them suitable to be used as biomaterials [7]. The strong glass former P_2_O_5_ contributes in the glass network, as PO_4_ structural units [6,8,9] are due to covalent bonding by the bridging oxygens. This bioglass material upon immersion in the simulated body fluid (SBF) solution shows a state where the ionic and pH conditions perfectly simulate with human blood plasma [7]. The ability to form rich calcium phosphate hydroxyapatite (HAp) layer on the surface of the bioglass samples when it comes into contact with the SBF confirms the in vitro bioactivity. The main disadvantage of the phosphate-based glasses is their poor mechanical strength, which limits the applications in implant development related to hard tissue replacement. This can be resolved by incorporating suitable transition metal ions, such as TiO_2_, MgO, ZnO, CuO, Fe_2_O_3_, etc., to phosphate glass network in appropriate amounts. The first generations of biomaterials are directly related to the bone and tissue engineering by the implantation of ZrO_2_ and TiO_2_-based materials [9,10,11]. The first research paper on zirconia was published by Helmer and Driskel in 1969 and mentioned that ZrO_2_ can be used as a biomaterial [12]. ZrO_2_ is one of the common trace elements present in the human body, and thus its inclusion in the bioglass could be exploited for stimulating bioactivity. Both the biological activity and the mechanical properties can improve with the incorporation of ZrO_2_ to the phosphate glass network. The phosphate glass structure is mainly strengthened by forming of Zr–O–P covalent bonds due to the entering of ZrO_6_ octahedra structural units of zirconia [13,14]. Most of the available research reports related to zirconia mixed silica based bioactive glasses revealed the gradual decrease in their biological activity with the increase of ZrO_2_ concentration. However, considerably limited work is available on ZrO_2_ contain phosphate based bioglasses and glass ceramics [15]. Zirconia mixed porous calcium titanium phosphate glass ceramic system was fabricated by V. K. Marghussian et al. and studied the effect of zirconia on chemical durability and mechanical strength, which are observed to be improved [15]. V. Rajendran et al., synthesized P_2_O_5_–Na_2_O–CaO–ZrO_2_ glasses with the addition of ZrO_2_ up to 1.0 mol% by replacing the Na_2_O and observed that considerable high bioactivity along with improved mechanical strength at 0.75 mol% of ZrO_2_ out of other concentrations [16]. Caiyun Zheng et al. prepared 60CaO–30P_2_O_5_–3TiO_2_–xZrO_2_–(7−x)Na_2_O (x = 0, 1, 3) glass ceramics and studied the influence of ZrO_2_ on mechanical and bioactive properties, and found that the toughening of the system with 1 mol% of ZrO_2_ added has no adverse effect on the bioactivity [17]. Contrary to earlier reports, we have considered that silica free zinc calcium phosphate glasses mixed with small quantities of ZrO_2_ might improve the structural and bioactivity suitable for tissue engineering implant applications. Moreover, the result of ZrO_2_ on structural and biological properties of the P_2_O_5_ glasses is not yet revealed completely. Therefore, ZrO_2_ doped bioactive glass system was prepared and analyzed to probe some light on structural and biological properties, by performing some experiments, such as XRD, FTIR, and SEM-EDS, etc., pre- and post-soaked in SBF, and monitoring the degradation and pH variation of the bioactive materials suitable for bone regeneration applications.

In this present study, we have developed the novel ZnO–Na_2_O–CaO–P_2_O_5_ bioglass system by doping less than 1 mol% of ZrO_2_, and explored the properties (thermal and structural) that stimulate the deposition of HAp layer over the glass surface.

## 2. Materials and Methods

### 2.1. Preparation of Bioglass Materials

The bioglass composition is given in Table 1. The ZrO_2_ mixed calcium phosphate glasses were synthesized by taking high purity (99.9%) P_2_O_5_, ZnO, CaO, Na_2_O and ZrO_2_ chemical compounds from Sigma-Aldrich (St. Louis, MO, USA) by the melt-quenching method. The details of the glass composition chosen for the present study and their corresponding codes are given in Table 1. The appropriate proportions of the chemicals (20 g batches) were homogeneously mixed in an agate mortar and melted in platinum crucibles at the temperature 1000 °C for 2 h. During melting, in order to ensure homogeneity, the melt was stirred for every half hour. The obtained melt was casted into pre-heated graphite molds and then shifted the glasses in an annealing chamber maintained at 250 °C; the annealing was carried out for 2 h and subsequently cooled at the rate of 1 °C/min to the room temperature, to make the samples free from the internal cracks, residual stress etc. The obtained samples were ground and well-polished to the final dimension of 1.5 cm × 1.5 cm × 0.2 cm.

### 2.2. Thermal Analysis

Thermal characterization was carried out on the glass powder by heating from room temperature to 1100 °C under Argon atmosphere, with a heating rate of 10 °C/min, using a standard NETZ5CH-STA 2500 (NETZSCH, Selb, Germany) Regulus thermal analysis system. A 20 mg of glass powder in an alumina pan is used for heating, with identical alumina pan as reference material. The obtained DTA patterns are used to identify the *T*_g_, *T*_c_, *T*_m_, and other thermal parameters, such as thermal stability (ΔT) and Hruby’s criterion (*K*_H_). The difference between *T*_c_ and *T*_g_ representing the thermal stability (ΔT) and the ration between *T*_c_ − *T*_g_ and *T*_m_ − *T*_c_ gives Hruby’s criterion (*K*_H_) of the glass system [18]:

(1) ∆T=Tc−Tg

(2) $$K_{H}=\frac{Tc-Tg}{Tm-Tc}$$

### 2.3. Bioactivity Assessment

The SBF is arranged in a polyethylene vessel by mixing appropriate quantities of analar grade reagents of NaCl, KCl, NaHCO_3_, MgCl_2_, 6H_2_O, CaCl_2_ and KH_2_PO_4_ (99.95%, Sigma-Aldrich) to distilled water with continuous stirring. The solution is buffered to pH 7.4 by adding Tris-buffer and hydrochloric acid is kept at 278 K for 48 h to trace the presence of any precipitates, the process recommended by Kokubo and Takadama [7]. After confirming the lack of precipitate, the obtained solution was used for in vitro studies. It was ensured that the ratios of concentration (mM) of different ions in the prepared solution were like those of human blood plasma (Table 2). In vitro bioactivity studies were performed (so as to achieve HAp layer formation on the surface of the samples) by immersing each sample (0.10g of glass powder) separately in 50 mL of SBF at 37 °C [19]. The weight loss measurements and the pH of the solution were performed after different incubation periods (viz. 0, 1 and 5 days).

### 2.4. Powder XRD

The structural phases of the glasses were analyzed by PANalytical X’pert Powder (Malvern Panalytical Ltd., Malvern, UK), using Cu-Kα as a radiation (λ = 1.540598 Ǻ) source. The XRD patterns were recorded diffraction angle range 2θ = 10–80°. Using the International Center for Diffraction Data cards (ISO 9001:2015 certified by DEKRA) were preferred to identify the crystal phases corresponding to each diffraction peak observed in the XRD pattern.

### 2.5. Fourier Transform Infrared Spectroscopy Analysis

The FTIR (model: S100, PerkinElmer, Shelton, CT, USA) transmittance spectra of the samples were recorded (for understanding the internal structural variations of the glass network) in the wavenumber range of 4000–400 cm^−1^. The mixture of 1 mg of glass powder and 300 mg of Potassium bromide powders was used to make pellets prepared under vacuum pressure.

### 2.6. SEM-EDS Micrographs

The surface morphology and microstructure of the bioglass samples were analyzed with the help of Scanning electron microscopy of VEGA 3 LMU, TESCAN (Brno, Czech Republic), pre- and post-immersion in SBF. The elemental analysis of the glass samples was estimated by Energy dispersive X-ray analyzer connected to SEM. In order to get clear images, the samples should be electroconductive; for this, the samples were coated with a thin gold layer.

### 2.7. Degradation Behavior

The dissolution behavior of the glasses immersed in SBF (pH 7.4 at 37.5 °C) was measured by a weight loss process. Initially, the powder samples were weighed pre immersion and then immersed for different days (0, 1 and 5) in SBF solution. Next, the glasses were removed from the solution and dried at 80 °C, and again measure the weight of each sample to determine the weight loss by the following:

(3) $$\mathrm{Weight loss}=\frac{Wo-Wt}{Wo}\times 100\%$$

where *W*_o_ is pre-immersion weight and *W*_t_ is post-immersion weight.

### 2.8. pH Evaluation

The pH values of the residual SBF were measured (so as to have the information on dissolution behavior of the bioactive glass) pre-and post-immersion of the samples, containing different contents of ZnO for different intervals of time by a pH meter (ORION pH 7000). The pH meter (Thermo Scientific, Beverly, MA, USA) pre-calibrated to 4.01, 7.00, and 9.20 was used for these measurements.

## 3. Results and Discussion

### 3.1. Thermal Properties

**Figure 1:**
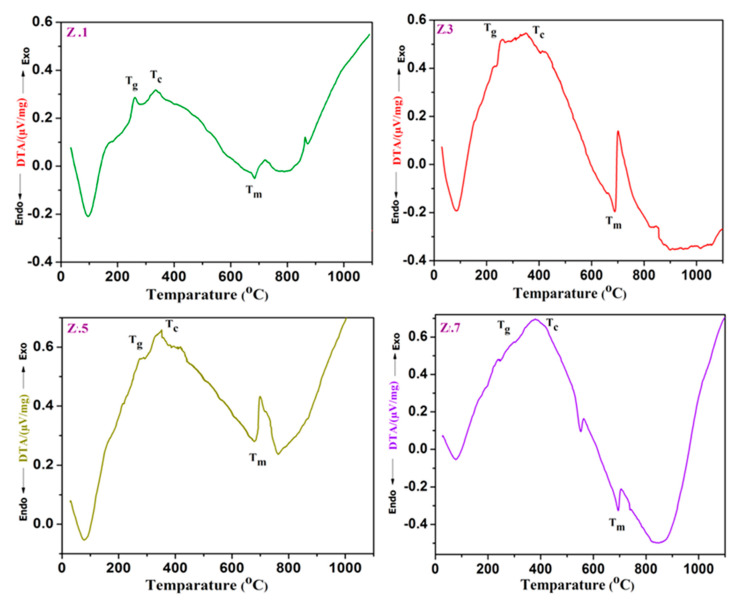
DTA analysis curves of ZrO_2_ doped bioglass samples Z.1, Z.3, Z.5 and Z.7.

**Figure 2:**
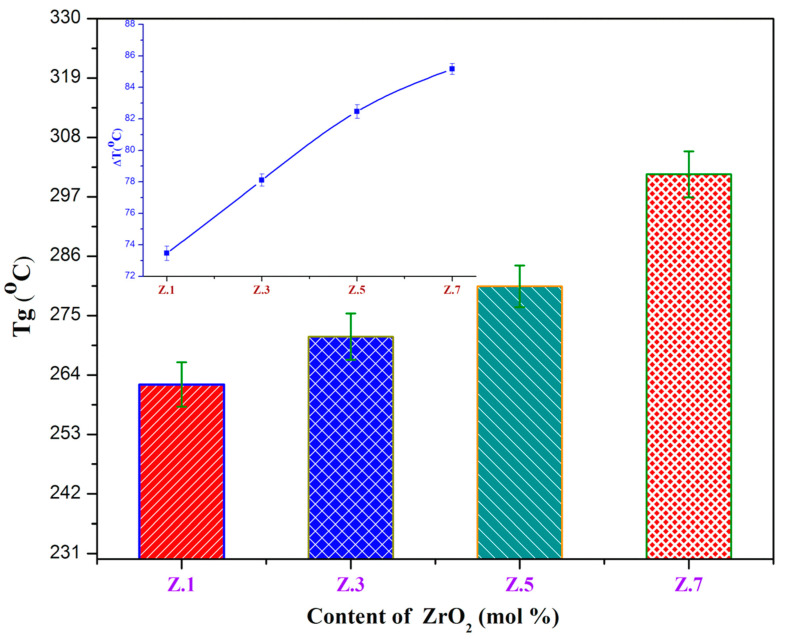
Variation of glass transition (*T*_g_) as a function of ZrO_2_ (mol%) content (inset shows the thermal stability vs ZrO_2_ concentration).

The traces of differential thermal analyses (DTA) are shown in Figure 1 and Figure 2, and the corresponding temperature values *T*_g_, *T*_c_, and *T*_m_ of ZrO_2_ doped glass samples are tabulated in Table 3. The values of glass transition temperature (*T*_g_) from 262.21 (± 1.11) °C to 301.15 (± 1.25) °C, crystallization temperature (*T*_c_) from 335.67 °C to 386.32 °C, and melting temperature (*T*_m_) from 672.67 °C to 697.49 °C increased with ZrO_2_ mol% (from 0.1 to 0.7). The increase in *T*_g_ values with an increase of zirconia is due to an increase in the average crosslink density through non-bridging oxygen ions (NBO) and the number of bonds per unit volume. In addition, an increase in *T*_g_ can also be due to the increasing aggregation effect of ZrO_2_ on the glass network and slow mobility of large Zr^4+^ ions, which lead to more rigidity of the glass network. The exothermic *T*_c_ and endothermic *T*_m_ peaks are also increased gradually with the addition of ZrO_2_ (of Zr^4+^ ions (0.72 A °)) [15]. Furthermore, it is found to raise the viscosity of glass with a gradual increase of zirconia content due to the decrement of NBO’s and/or high ionic field strength [1,3,11].

In the current ZrO_2_ doped glasses, the glass transition temperature (*T*_g_) and stability (ΔT (°C)) values increased from 262.21 ± 1.11 to 301.15 ± 1.25 and from 73.46 ± 0.45 °C to 85.16 ± 0.35 °C respectively, whereas the Hruby criterion (*K*_H_) increased from 0.22 to 0.27 with the content of ZrO_2_, which undoubtedly designates the high stability and the good glass-forming tendency of as-prepared glasses. The obtained results confirm the structural modification and thermal stability of the zirconia incorporated bioactive glasses.

### 3.2. XRD Analysis

**Figure 3:**
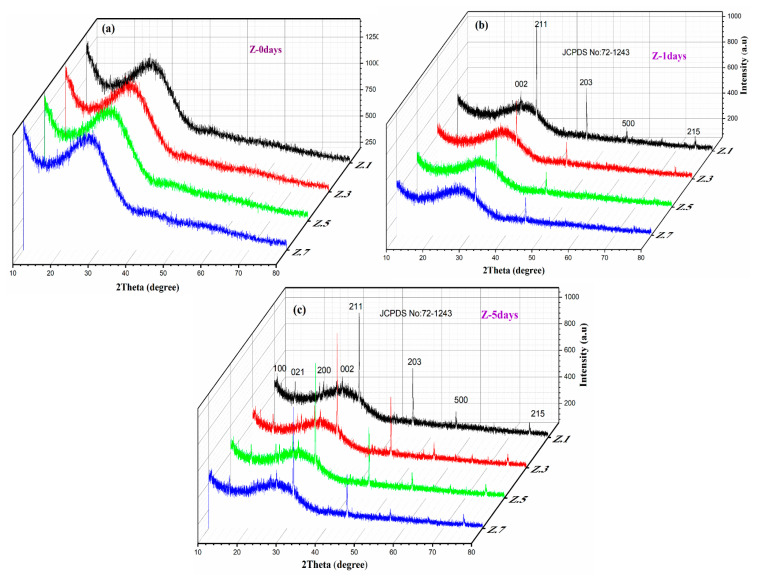
X-ray diffraction patterns of the bioglass samples after immersion in SBF (**a**) 0 day, (**b**) 1 day and (**c**) 5 days.

Figure 3 illustrates the X-ray diffractograms of ZrO_2_ mixed phosphate glasses immersed in SBF in 0, 1 and 5 days of intervals recorded at the diffraction angle (2θ) in between 10–80°. Before immersion, the samples do not show any sharp crystalline peaks, which indicate the non-crystalline nature of the samples [8] shown in Figure 3a. After immersion, the same samples exhibited prominent crystalline peaks; this indicates the formation of hydroxyapatite layer (HAp: Ca_10_(PO_4_)_6_(OH)_2_) on the surface of the glass samples, due to ion leaching from glass to SBF and vice versa. The intensity reflections at (1 0 0), (0 2 1), (2 0 0), (0 0 2), (2 1 1), (2 0 3), (5 0 0), and (2 1 5), in accordance with (h k l) values, are represented formation of the hydroxyapatite layer on the glass surface samples. These intensity reflections of HAp from XRD patterns were indexed using JCPDS card No: 72-1243. After soaking for 1 day (Figure 3b), the presence of intense diffraction peaks at 31.74° (2 1 1) and 45.32° (2 0 3) in the XRD diffractograms indicates the growth of crystalline calcium phosphate hydroxide [18,19]. With the increasing immersion time (1 day to 5 days), additional peaks are appearing laterally with the noticeable intense peaks existing during 1 day of incubation. This is predicted due to penetration of Ca^2+^ ions into PO_4_^3−^ glass network leading to the formation of a crystalline HAp layer on glass surface [3,10]. Moreover, it is clearly noticed that the peak intensities are increased with the incubation time and decreased with the concentration of ZrO_2_, due to the dissolution kinetics of the glass in SBF solution. This deposition of thin HAp layer over the glasses soaked in physiological fluid can be directly correlated to their capacity to generate effective chemical bonds with natural bone tissues [20,21]. The attained results from XRD on bioactivity of as prepared bioglasses are also confirmed further by FTIR and SEM studies.

### 3.3. FTIR Spectroscopic Analysis

**Figure 4:**
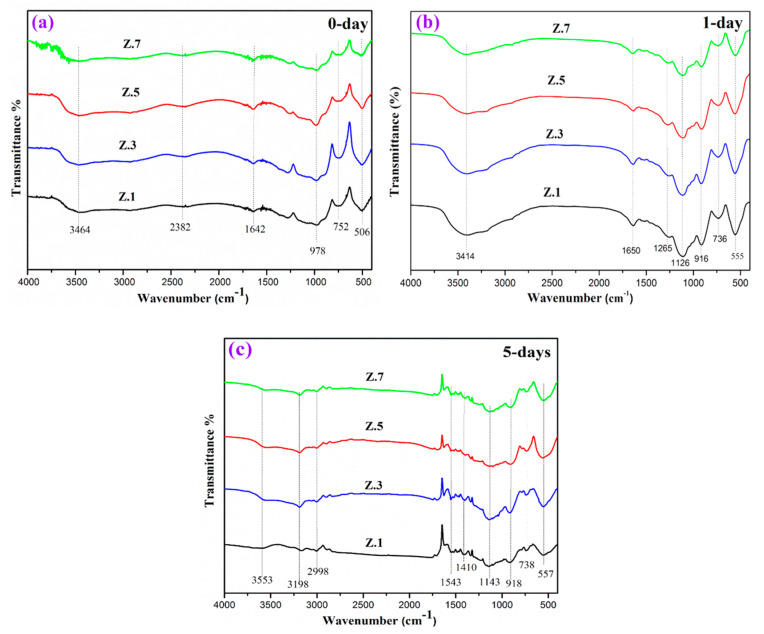
FTIR absorption spectra from the glass surface: (**a**) 0 day before and after (**b)** 1 day, (**c**) 5 days’ immersion in several days in SBF.

Figure 4 demonstrates the infrared transmission spectrum of zirconia doped phosphate samples pre and post immersion in SBF, and the assignment of various bands presented in Table 4. The FTIR spectra of before soaked samples (Figure 4a) are shown in the characteristic phosphate bands located at 506 cm^−1^, 752 cm^−1^, 978 cm^−1^, 1642 cm^−1^, 2382 cm^−1^ and 3464 cm^−1^. The band at around 506 cm^−1^ is attributed to the bending vibrations (PO_4_^3−^) of O–P–O [15,16,22] The absorption bands at 752 cm^−1^ and 978 cm^−1^ are due to the (P–O–P) symmetric stretching vibrations of phosphate group [10,23]. The peak observed at 1642 cm^−1^ is assigned due to the stretching vibrations of P–O–H groups (water molecule) [9]. A minor peak at around 2382 cm^−1^ is due to CO_3_^2−^ and HCO_3_^−^ groups [23,24] and a wide intense band at 3464 cm^−1^ is ascribed to the symmetric stretching of O–H groups [9].

After immersion of 1 day and 5 days (Figure 4b,c) in SBF, the spectra showed the presence of additional bands (phosphate structural group) 557 cm^−1^, 736–738 cm^−1^, 916–918 cm^−1^, 1126–1143 cm^−1^, 1263 cm^−1^, 1652 cm^−1^, 1543 cm^−1^, 2998 cm^−1^, 3198 cm^−1^, 3414 cm^−1^, 3553 cm^−1^, along with the bands before incubation in SBF. After immersion, the band at 555-557 cm^−1^ represents the HAp typical bond (PO_3_^4^) of a phosphate group and due to the hydroxyapatite crystallization [25]. The medium bands 736–738 cm^−1^ (P–O–P) can be assigned to the pyrophosphate (P_2_O_7_)^4−^ group and the bands at around 916–918 cm^−1^ are corresponding to the P–O–P stretching vibrations [26]. The strong bands appearing at 1126–1143 cm^−1^ are attributed to the PO_2_ symmetric stretching vibration, which is related to the calcium phosphate surface layer. The lower band at 1263 cm^−1^ is assigned to P=O stretching vibrations/anti-symmetrical vibrations of PO_2_^–^ groups. The 1414 cm^−1^ band is –OH hydroxyl carbonate group, while the band noticed at 1543 cm^−1^ band is the deformation of –OH groups [22,25,27].

The bands appearing between 1642–1650 cm^−1^ are due to stretching vibrations of P–O–H group [28] and the bands around 3414–3464 cm^−1^ and 3553 cm^−1^ are ascribed to the O–H symmetric stretching [25,29]. The band at 2382 cm^−1^ is assigned to the O–H stretching vibrations of hydrogen-bonded H–O–H groups on the surfaces of the sample. Very small peaks could also be observed at 2998 cm^−1^ and 3198 cm^−1^ due to stretching vibrations in C–H groups [22,29,30]. The entrance of PO_3_^2–^, CO_3_^2−^ and OH groups present in the FTIR spectra of the glasses confirmed the growth of the thin crystalline HCA layer. It was also observed that the intensities of the absorption peaks increased with an increase of soaking time, as well as the zirconia inclusion in the glass network [15,31]. Moreover, obtain results correlated with the XRD results. Furthermore, the formation of the HAp layer was confirmed by the SEM-EDS results.

### 3.4. SEM-EDS Analysis

**Figure 5:**
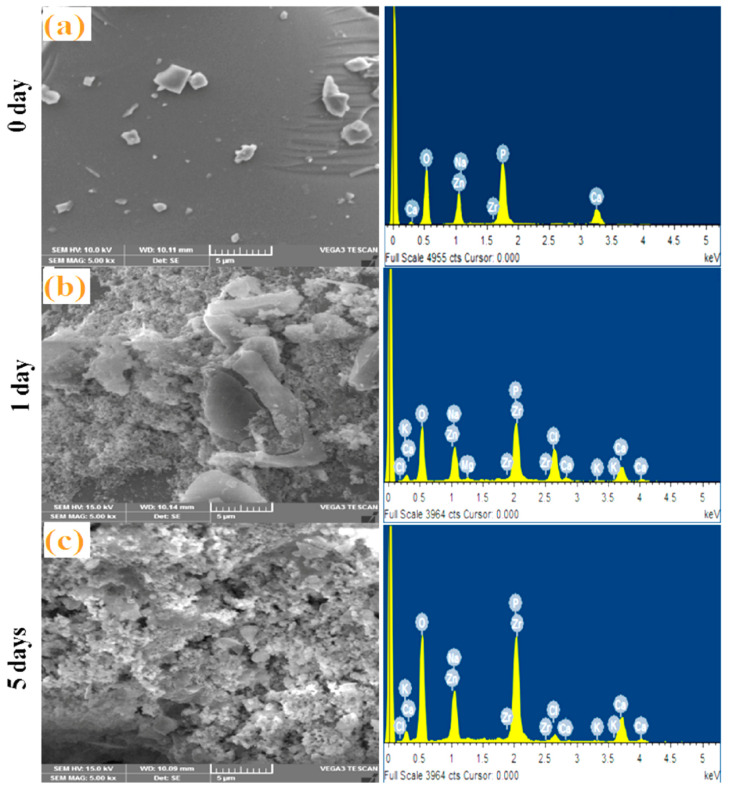
SEM micrograph and EDS analysis of the bioglass sample Z.5 magnifications: for (**a**) 0 day before and (**b**) 1 day, (**c**) 5 days after immersion in SBF solution.

Figure 5 displays the surface morphology of bioglasses pre- and post-immersion in SBF by SEM-EDS analysis. The micrographs reveal the precipitation over the samples after incubation, indicating the formation of the hydroxyapatite (HAp) layer. Morphologies of the Z.5% (Figure 5a) glass sample before incubation (0-day) in SBF visualize the plane surface by SEM and existence of minimal elements P, Ca, Zn, Na, Zr and O of the glass by EDS, clearly indicating their amorphous nature and lack of any precipitation formation of the samples [6]. After 1 day and 5 days of immersion in SBF, the bioglass surface shows changes in surface morphology (cotton-like structures) and creates the appearance of additional elements (P, Ca, Zn, Na, Zr, O, Cl, K, Mg) besides the authentic glass compositional elements, indicating the deposition of apatite layer and therefore, it can be concluded that the prepared bioglass samples are bioactive. After 1 day, as observed in Figure 5b, the SEM images exhibited small concentrations of precipitation, as the crystalline nature of the apatite layer is identified from XRD analysis. SEM images also have been taken for samples after 5 days of immersion, where the apatite layer on the glass sample surfaces are noticed to be fully covered, and the dense HAp layer (and also from EDS Ca-P ratios data) has been developed (~39 microns thick) [32]. In addition, there is an increment in the Ca and P intensity peaks from EDS after immersion in SBF. The obtained Ca/P ratios of the synthesized glasses are changing from 1.45–1.68 and are very near to human bone Ca/P ratios [12,33]. This is mainly because of Ca^2+^, Na^+^ and phosphate ions releasing from glass surface to SBF solution and transfer of ions from solution to the glass surface. Moreover, it leads to the easy super saturation of Ca^2+^ and PO_4_^3−^ ions on the surface of glasses and is favorable for HAp to nucleate and grow [7]. These obtained results are in good agreement with the above mentioned XRD and FTIR data.

### 3.5. pH Measurement and Weight Loss Studies

**Figure 6:**
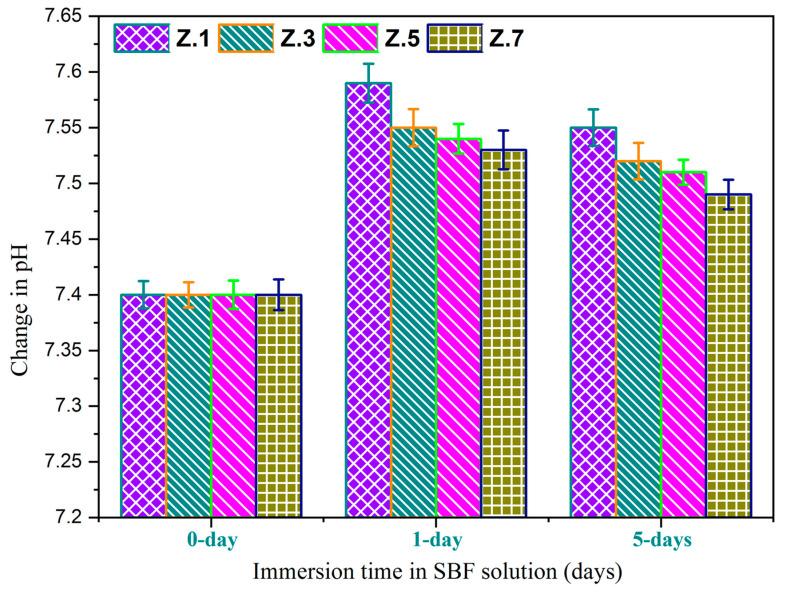
pH variation for all the bioglass samples after immersion in SBF solution.

**Figure 7:**
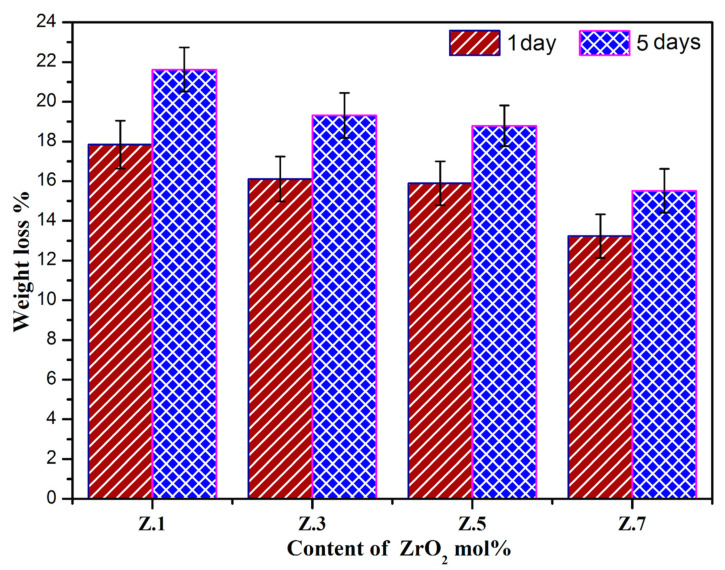
Weight loss of ZrO_2_ doped glasses after immersion in SBF for 1 day and 5 days.

From Figure 6, it can be noticed that the pH values are increased for after immersion in the SBF solution for 1 day suddenly, while after, there is a gradual reduction of the pH values for five days of immersion time. The initial raise of pH values of the residual SBF (during the immersion from 0 to three days) is due to the release of alkali/alkaline ions (viz., Na^+^, or Ca^2+^) and even the migration of zirconium species into the SBF that causes the increase of the basicity of SBF. The detected pH reduction in the SBF with the rise in the soaking period is owing to the formation of more concentrated phosphoric acid in SBF and might be the transfer of alkaline Ca^2+^ ions from SBF to the surface of the sample (to form HAp layer) [34]. Moreover, it is observed that the pH of the residual SBF decreases slightly with the increase in content of ZrO_2_ and obeys the same trend as that of degradation. Figure 7 illustrates the weight loss of glass samples upon SBF treatment for different time intervals (1 day and 5 days). With an increase in the immersion time, the gradual degradation of glass samples also increased. Therefore, the accurate dissolution rate of Z.1 to Z.7 glass samples’ increments is based on the Ca^2+^ and PO_4_^3−^ ions from samples, due to their dissolution in the SBF solution. As we can see, the weight loss of each bioglass increased along with the immersion time, but decreases slightly by adding of ZrO_2_ up to 0.7 mol% in the phosphate glass matrix and resemble earlier reported literature [35,36]. The slight decrease in the rate of degradation along with increasing ZrO_2_ content is observed, which is mainly because of highly established cross-linked dense structures and a reduction of the degradation of the glass. This result implies a strengthening network with the addition of ZrO_2_ to phosphate glasses [37,38]. These considerable changes occurred in pH, and the weight loss of as-developed bioglasses is necessary for the development of bone-like apatite.

## 4. Conclusions

ZrO_2_ doped calcium phosphate bioactive glasses were successfully synthesized through the melt-quenching technique. Thermal parameters such as glass stability (ΔT) and Hruby criterion (*K*_H_) values increase with the content of zirconia, which describes the high stability and the good glass-forming tendency of as-prepared glasses. The formation of the hydroxyapatite layer was confirmed by structural studies by means of XRD, FTIR, and SEM. The ratio of Ca and P from EDS is around 1.67, which is almost equal to bone composition and the ability to produce bone-like apatite structures on the surface. The changes that occurred in pH and weight loss of bioglasses with immersion time and zirconia content are desirable for the formation of bone-like apatite. In vitro studies revealed that the ZrO_2_ incorporated phosphate glasses exhibit high bioactivity relevant for bone tissue engineering applications.

## Figures and Tables

**Table 1:** The Nominal bioactive glass (mol%) composition.

Glass Code	ZnO	Na_2_O	CaO	P_2_O_5_	ZrO_2_
Z.1	8.0	22.0	23.9	46.0	0.1
Z.3	8.0	22.0	23.7	46.0	0.3
Z.5	8.0	22.0	23.5	46.0	0.5
Z.7	8.0	22.0	23.3	46.0	0.7

**Table 2:** The concentration of various ions in the simulated body fluid (SBF) solution.

Ion Type	Na^+^	K^+^	Mg^2+^	Ca^2+^	Cl^−^	HCO^3−^	HPO_4_^2−^	SO_4_^−^
Concentration (mM)	142.0	5.0	1.5	2.5	148.8	4.2	1.0	0.5
Human blood plasma	142.0	5.0	1.5	2.5	103.0	27.0	1.0	0.5

**Table 3:** Thermal properties of the ZrO_2_ containing bioglasses.

Sample Code	*T*_g_ (°C)	*T*_c_ (°C)	*T*_m_ (°C)	ΔT (°C)	*K*_H_
**Z.1**	262.21 (± 1.11)	335.67	672.67	73.46 (± 0.45)	0.22
**Z.3**	271.10 (± 1.30)	349.21	686.92	78.10 (± 0.38)	0.23
**Z.5**	280.40 (± 1.24)	362.87	691.84	82.47 (± 0.43)	0.25
**Z.7**	301.15 (± 1.25)	386.32	697.49	85.16 (± 0.35)	0.27

**Table 4:** Assignments of various bands from FTIR spectra of the bioglasses.

Wavenumber (cm^−1^)			Assignments	References
0 day	1 day	5 days		
506	555	557	~506 PO_4_^3−^ O–P–O bending vibrations/P–O amorphous	[15,16,22]
			~555–557 HAp (PO_3_^4^)	[25]
752	736	738	~752 P–O–P symmetric stretching ~736–738 P–O–P pyrophosphate (P_2_O_7_)^4−^ group	[10,23] [26]
978	916	918	~978cm^−1^ P–O–P stretching vibrations ~916–918 P–O–P stretching vibrations	[10,23] [26]
-	1126	1143	PO_2_ symmetric stretching vibration	[22,25,27]
-	1265	-	PO_2_^−^ asymmetric group /P=O stretching vibration	[22,25,27]
-	-	1410	–OH, hydroxyl carbonate group	[22,25,27]
-	-	1543	–OH groups	[22,25,27]
1642	1650	-	~1642–1650 cm^−1^ stretching vibrations of P-O-H group	[9,28]
2382	-	-	P–O–H group /CO_3_^2−^ and HCO_3_^−^ groups	[23,24]
-	2998	-	C–H stretching vibrations	[22,29,30]
-	-	3198	C–H	[22,29,30]
3464	3414	3553	~3414–3464 H–O–H bond /CO_3_^2−^ and HCO^3−^	[9,25,29]
			~3553 O–H symmetric stretching	[25,29]
